# Design and Implementation of a GaN-Based Three-Phase Active Power Filter

**DOI:** 10.3390/mi11020134

**Published:** 2020-01-24

**Authors:** Chao-Tsung Ma, Zhen-Huang Gu

**Affiliations:** Department of Electrical Engineering, CEECS, National United University, Miaoli 36063, Taiwan; M0621002@smail.nuu.edu.tw

**Keywords:** gallium nitride (GaN), power switching device, active power filter (APF), power quality (PQ)

## Abstract

Renewable energy (RE)-based power generation systems and modern manufacturing facilities utilize a wide variety of power converters based on high-frequency power electronic devices and complex switching technologies. This has resulted in a noticeable degradation in the power quality (PQ) of power systems. To solve the aforementioned problem, advanced active power filters (APFs) with improved system performance and properly designed switching devices and control algorithms can provide a promising solution because an APF can compensate for voltage sag, harmonic currents, current imbalance, and active and reactive powers individually or simultaneously. This paper demonstrates, for the first time, the detailed design procedure and performance of a digitally controlled 2 kVA three-phase shunt APF system using gallium nitride (GaN) high electron mobility transistors (HEMTs). The designed digital control scheme consists of three type II controllers with a digital signal processor (DSP) as the control core. Using the proposed APF and control algorithms, fast and accurate compensation for harmonics, imbalance, and reactive power is achieved in both simulation and hardware tests, demonstrating the feasibility and effectiveness of the proposed system. Moreover, GaN HEMTs allow the system to achieve up to 97.2% efficiency.

## 1. Introduction

In recent years, the rapid increase in the penetration level of renewable energy (RE)-based distributed power generation (DPG) has resulted in noticeable degradation in the voltage stability and power quality (PQ) of existing power systems. The intrinsic features of DPG systems include (1) intermittent power flow caused by the utilization of maximum power point tracking (MPPT) control functions and (2) harmonic current injections caused by various power converters using switching type control techniques. Moreover, modern technologies such as automatic manufacturing systems (AMS), Internet of Things (IoT), and Industry 4.0 require a large number of power converters based on high-switching frequency power electronic devices. It can be imagined that the combination of a variety of different harmonics in load currents and unbalanced active and reactive powers can negatively affect the PQ of power networks. In practice, there are a number of compensating schemes and devices commonly used for PQ improvement applications, such as capacitor banks, passive filters, series active power filters, shunt active power filters and their combinations. Of the reported methods, the active power filter (APF) is a very widely adopted solution because, with appropriate control strategy, it is capable of providing simultaneous compensation for voltage sag, harmonic current, imbalance, and active and reactive powers. Moreover, with an external energy source and/or energy storage devices, it can also be used as an uninterruptable power supply (UPS). The main unit of an APF system is a power converter that performs the desired PQ control functions though proper switching control of power electronic devices. An APF is usually connected to the point of common coupling (PCC) in parallel or series to perform its designed functions; however, shunt APFs are more commonly used because they provide more flexible compensation functions though current-injecting control and require less auxiliary equipment. When a series APF and a shunt APF are connected with a common direct current (DC) link, they can be used simultaneously, known as a unified power quality conditioner (UPQC) [1,2,3,4].

In open literature, there are a lot of papers investigating the application of APF to the mitigation of PQ issues, such as the improvement of PCC voltage stability [5,6], compensation of harmonic currents [5,6,7] and unbalance load currents [7,8], injection of active power [8,9], regulation of reactive power [6,9], etc. In order to develop high-performance APFs, it is crucial to improve the switching performance of power converters. In other words, it is very desirable to achieve higher efficiency, shorter response time, and higher power density of power converters used in APFs. To realize the aforementioned objective, wide-bandgap (WBG)-based power switching devices offer a promising solution. Gallium nitride (GaN) is a widely discussed WBG semiconductor material that benefits power-switching device technology with higher voltage, higher switching frequency, higher power, and better high-temperature capability in power switches compared with conventional silicon (Si)-based technologies. It has been expected that GaN high electron-mobility transistors (HEMTs) can greatly enhance the performance of power converters with less than 1-kV power switching requirement [10,11,12]. However, in open literature, the published papers on GaN HEMTs mostly address the manufacturing, device characteristics, driving, and switching performances [13]. Only two papers regarding GaN-based single-phase APFs were found in the Institute of Electrical and Electronics Engineers (IEEE)/Institution of Engineering and Technology (IET) Electronic Library (IEL) and ScienceDirect OnSite (SDOS) databases [14,15]. In [14], a GaN-based single-phase APF with a modified sigma-delta modulator technique was proposed and verified with simulation studies. In [15], a 5kW single-phase hybrid APF with a new control system was proposed to improve the system performance. There are currently few papers addressing the design issues and reporting performances of GaN-based three-phase inverters in practical application cases. As a result, this paper presents the key design procedure and demonstrates for the first time the performance of a 2-kVA GaN-based three-phase shunt APF system.

Following the introduction in this section, the next section will briefly describe the features of GaN HEMTs and its driving requirements. The third section addresses the mathematical modeling and control strategy of the proposed GaN-based three-phase APF based on synchronous reference frame (SRF) theory. In the fourth section, the proposed APF and control system are simulated using a comprehensive PQ control scenario. Hardware implementation and a test of a 2 kVA prototype are carried out in the fifth section. The sixth section provides some discussions on key technical issues related to the proposed GaN based three-phase APF. Finally, this paper is concluded in the last section.

## 2. Gallium Nitride (GaN) High Electron-Mobility Transistor (HEMT) and Its Driving Requirements

GaN HEMTs are believed to be the most promising solution for low- to medium-power applications because of their advantages such as higher breakdown voltage, lower on-resistance, and higher switching speed compared with conventional Si-based switching devices; these advantages can increase system efficiency and power density significantly and thus lead to new opportunities for achieving power converters with improved performance. Commercially available GaN HEMTs now achieve up to 650 V/50 A and 900 V/15 A [13]. 

There are normally on and normally off GaN HEMTs. Normally on GaN HEMTs, also known as depletion mode (D mode) GaN HEMTs, are not popular because normally off switching devices are a common requirement for power converter applications. On the other hand, normally off GaN HEMTs such as enhancement mode (E mode) GaN HEMTs and cascode GaN HEMTs can be turned on with positive *V_GS_* and turned off with zero or negative *V_GS_*. Generally, the turn-on threshold and highest allowed driving voltages of a GaN HEMT are much smaller than those of conventional Si-based switches. As a result, the careful design of driving circuit is necessary in order to avoid fault turn-on and high overshoot. Common suggestions include providing separate turn-on and turn-off driving paths, achieving minimized overlapping between driving and power loops, using Miller clamp and negative voltage sources to ensure reliable turn-offs, etc. [13].

## 3. Mathematical Modeling and Control Algorithms of GaN-Based Active Power Filter (APF)

### 3.1. GaN-Based Three-Phase Active Power Filter

**Figure 1:**
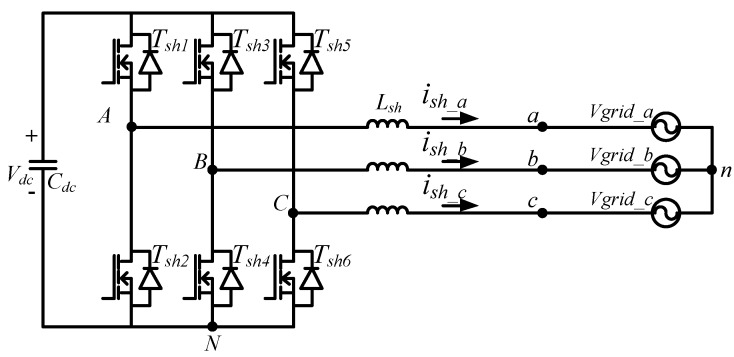
Shunt three-phase inverter.

The main power electronic circuit in a three-phase shunt APF system is a three-phase inverter. The main control functions in an APF are to adjust the DC link voltage of the three-phase inverter to the rated value and to compensate reactive power, unbalanced current and harmonic components of the load as required. The circuit architecture of the proposed three-phase inverter is shown in Figure 1. The DC link voltage control adopts dual-loop control schemes, where the inner loop controls inductor currents, and the outer loop controls DC link voltage. By controlling the inductor currents, the goals of regulating DC link voltage and compensating harmonics, unbalanced and reactive components of load currents can be achieved.

The circuit specifications of the proposed three-phase inverter developed in this paper are the following: the three-phase line to line voltage of the grid = 110 V_rms_, grid frequency = 60 Hz, rated power = 2 kVA, DC link voltage = 200 V, switching frequency = 50–100 kHz, DC voltage sensing factor = 0.012, AC current sensing factor = 0.05, AC voltage sensing factor = 0.0062, and DC voltage variation limit = 1%. 

### 3.2. Design of Direct Current (DC) Capacitor and Filter Inductors

The main function of the DC link capacitor is to stabilize DC link voltage. If the DC link capacitance is too large, the dynamic response of the DC link voltage will be slow, and the cost of the APF hardware system will be increased; if the DC capacitor is too small, it will be difficult to suppress the disturbance caused by external power flow. In order to design the appropriate size of the capacitor, we first define instantaneous power of the DC link:

(1) $$P_{dc}=V_{dc}I_{dc}=({\bar{V}}_{dc}+{\tilde{V}}_{dc})({\bar{I}}_{dc}+{\tilde{I}}_{dc}),$$

where *V_dc_* and *I_dc_* represent DC voltage and current, respectively, which can both be separated into their respective DC components (${\bar{V}}_{dc}$ and ${\bar{I}}_{dc}$) and AC components (${\tilde{V}}_{dc}$ and ${\tilde{I}}_{dc}$). In order to simplify the analysis, we make three assumptions: the conversion efficiency of the three-phase inverter is 100%, ${\tilde{V}}_{dc}$ is considered zero, and ${\bar{I}}_{dc}$ is considered zero because ${\tilde{I}}_{dc}$ is generally far larger than ${\bar{I}}_{dc}$. As a result, we obtain the following:

(2) $$P_{dc}≅{\bar{V}}_{dc}{\tilde{I}}_{dc}(t)={\bar{V}}_{dc}C_{dc}\frac{d{\tilde{V}}_{dc}(t)}{dt},$$

where *C_dc_* represents DC link capacitance. Then, we obtain the voltage variation of the DC link capacitor:

(3) $${\tilde{V}}_{dc}(t)=\frac{1}{C_{dc}{\bar{V}}_{dc}}\int_{0}^{t}P_{dc}(t)dt,$$

where $\int_{0}^{t}P_{dc}(t)dt$ represents the capacity of the three-phase inverter. Then, we obtain DC link capacitance:

(4) $$\Delta V_{dc}=\frac{S}{f_{sw}C_{dc}{\bar{V}}_{dc}}\Rightarrow C_{dc}=\frac{S}{f_{sw}\Delta V_{dc}{\bar{V}}_{dc}},$$

where *f_sw_* represents the switching frequency. It should be noted that if an electrolytic capacitor were used for this APF design case, a higher capacitor specification will be required.

The function of the filter inductors is to filter out current ripples caused by the switching of the shunt three-phase inverter. Large inductances suppress the ripples of inductor currents but reduce the response speed of current controllers. On the other hand, although small inductances improve the response speed of the current controller, they cause large current ripples. Therefore, the inductances can be adjusted according to the actual situation. In order to design the filter inductances, we first need the following inductor voltage equation:

(5) $$v(t)=L_{sh}\frac{di_{sh}(t)}{dt},$$

where *L_sh_* represents inductance value, and *i_sh_* represents inductor current. According to the relationship between voltage and current on an inductor, (5) can be expressed as follows.

(6) ΔIsh=D2×Tsw×(Vdc−Vgrid)Lsh,

where Δ*I_sh_* represents shunt inductor current ripple, *D* represents duty cycle, *T_sw_* represents switching period, and *V_grid_* represents grid voltage. The duty cycle can be expressed the following:

(7) $$D(\omega t)=m_{a}\sin(\omega t),$$

where *m_a_* represents modulation factor and equals modulation signal divided by triangular wave amplitude (*v_con_*/*v_tri_*). Then, we get output AC voltage:

(8) $$V_{sh}(\omega t)=V_{dc}m_{a}\sin(\omega t).$$

Substituting (7) and (8) into (6) yields the following:

(9) $$\Delta I_{sh}=\frac{V_{dc}\times T_{sw}}{2L_{sh}}m_{a}\sin(\omega t)\left[1-m_{a}\sin(\omega t)\right].$$

Then, we differentiate (9) and let the result be zero in order to obtain the maximum value of inductor current ripple:

(10) $$\frac{d\Delta I_{sh}(\omega t)}{d\omega t}=\frac{V_{dc}T_{sw}}{2L_{sh}}m_{a}\left[\cos(\omega t)-2m_{a}\sin(\omega t)\cos(\omega t)\right]=0.$$

As a result,

(11) $$\sin(\omega t)=\frac{1}{2m_{a}}.$$

Lastly, substituting (11) into (9) yields the following equation:

(12) $$L_{sh}=\frac{V_{dc}}{8f_{sw}\Delta I_{sh}}.$$

According to the circuit specifications of the three-phase inverter and commonly assumed inductor current ripple, 10% of output current, it is calculated that the required inductance should be at least larger than 500 μH.

### 3.3. Mathematical Modeling and Controller’s Design for GaN-Based Shunt APF

#### 3.3.1. Mathematical Modeling

The mathematical model of the shunt-connected three-phase inverter can be derived according to Figure 1. First, the following equations are obtained with Kirchhoff’s voltage law:

(13) $$L_{sh}\frac{di_{sh\_a}}{dt}=v_{AN}-v_{grid\_a}-v_{nN},$$

(14) $$L_{sh}\frac{di_{sh\_b}}{dt}=v_{BN}-v_{grid\_b}-v_{nN},$$

(15) $$L_{sh}\frac{di_{sh\_c}}{dt}=v_{CN}-v_{grid\_c}-v_{nN},$$

where *i_sh_a_*, *i_sh_b_*, and *i_sh_c_* represent three-phase inductor currents, *v_AN_*, *v_BN_*, and *v_CN_* represent switching point voltages, *V_grid_a_*, *V_grid_b_*, and *V_grid_c_* represent three-phase grid voltages, and *v_nN_* represents the voltage between the grid ground and the inverter ground. Also, the three-phase three-wire system satisfies the following condition:

(16) $$i_{sh\_a}+i_{sh\_b}+i_{sh\_c}=0.$$

As a result, *v_nN_* can be expressed as the following:

(17) $$v_{nN}=\frac{\left(v_{AN}+v_{BN}+v_{CN})-(v_{grid\_a}+v_{grid\_b}+v_{grid\_c}\right)}{3}.$$

Substituting Equation (17) into Equations (13)–(15) yields the following:

(18) $$\left[\begin{array}{l} L_{sh}\frac{di_{sh\_a}}{dt}\\ L_{sh}\frac{di_{sh\_b}}{dt}\\ L_{sh}\frac{di_{sh\_c}}{dt} \end{array}\right]=\frac{2}{3}\left[\begin{array}{l} 1\frac{-1}{2}\frac{-1}{2}\\ \frac{-1}{2}1\frac{-1}{2}\\ \frac{-1}{2}\frac{-1}{2}1 \end{array}\right](\left[\begin{array}{l} v_{AN}\\ v_{BN}\\ v_{CN} \end{array}\right]-\left[\begin{array}{l} v_{grid\_a}\\ v_{grid\_b}\\ v_{grid\_c} \end{array}\right]).$$

In this study, pulse width modulation (PWM) is used in the control, where the three-phase modulation signals *v_cona_*, *v_conb_*, and *v_conc_* are compared with *v_tri_* respectively to trigger the switches of all three switching legs. The output voltages of the switching legs can be expressed as follows:

(19) $$v_{aN}=(\frac{1}{2}+\frac{v_{cona}}{2v_{tri}})V_{dc};$$

(20) $$v_{bN}=(\frac{1}{2}+\frac{v_{conb}}{2v_{tri}})V_{dc};$$

(21) $$v_{cN}=(\frac{1}{2}+\frac{v_{conc}}{2v_{tri}})V_{dc}.$$

Substituting Equations (19)–(21) into Equation (18) and letting *V_dc_*/2*V_tri_* = *K_pwm_* yield the following:

(22) $$\left[\begin{array}{l} L_{sh}\frac{di_{sh\_a}}{dt}\\ L_{sh}\frac{di_{sh\_b}}{dt}\\ L_{sh}\frac{di_{sh\_c}}{dt} \end{array}\right]=\frac{2}{3}\left[\begin{array}{l} 1\frac{-1}{2}\frac{-1}{2}\\ \frac{-1}{2}1\frac{-1}{2}\\ \frac{-1}{2}\frac{-1}{2}1 \end{array}\right](K_{pwm}\left[\begin{array}{l} v_{cona}\\ v_{conb}\\ v_{conc} \end{array}\right]-\left[\begin{array}{l} v_{grid\_a}\\ v_{grid\_b}\\ v_{grid\_c} \end{array}\right]).$$

Using SRF theory, Equation (22) can be converted into the following:

(23) $$\left[\begin{array}{c} L_{sh}\frac{dI_{sh\_d}}{dt}\\ \begin{array}{l} L_{sh}\frac{dI_{sh\_q}}{dt}\\ L_{sh}\frac{dI_{sh\_0}}{dt} \end{array} \end{array}\right]=K_{pwm}\left[\begin{array}{ccc} 1 & 0 & 0\\ 0 & 1 & 0\\ 0 & 0 & 1 \end{array}\right]\left[\begin{array}{c} v_{cond}\\ \begin{array}{l} v_{conq}\\ v_{con0} \end{array} \end{array}\right]-\left[\begin{array}{ccc} 1 & 0 & 0\\ 0 & 1 & 0\\ 0 & 0 & 1 \end{array}\right]\left[\begin{array}{c} V_{grid\_d}\\ \begin{array}{l} V_{grid\_q}\\ V_{grid\_0} \end{array} \end{array}\right]-\left[\begin{array}{ccc} 0 & \omega L_{sh} & 0\\ -\omega L_{sh} & 0 & 0\\ 0 & 0 & 0 \end{array}\right]\left[\begin{array}{l} I_{sh\_d}\\ I_{sh\_q}\\ I_{sh\_0} \end{array}\right].$$

#### 3.3.2. Design of Current Controllers

**Figure 2:**
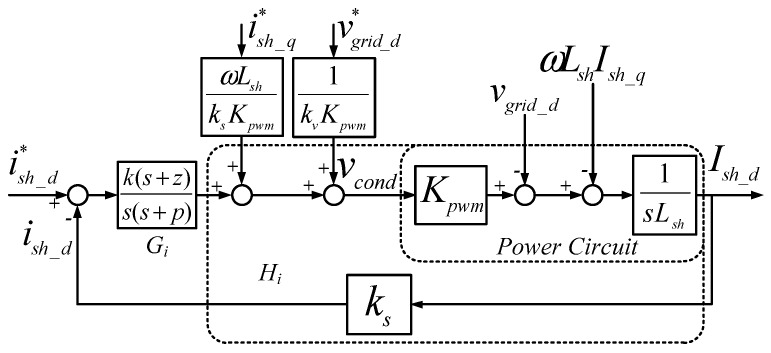
Block diagram of d-axis current controller.

**Figure 3:**
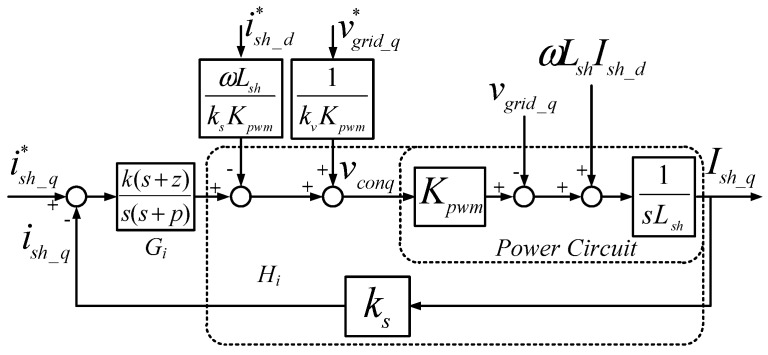
Block diagram of q-axis current controller.

According to Equation (23), we can obtain block diagrams of direct-quadrature axis (d-q axis) current loops with type-II controllers as shown in Figure 2 and Figure 3, where *k_s_* and *k_v_* represent AC current and voltage-sensing factors, respectively. Under ideal feed-forward conditions, the transfer function of current loop (d-axis or q-axis) is as follows:

(24) $$H_{i}(s)=\frac{k_{s}K_{pwm}}{sL_{sh}}.$$

The transfer function of the adopted type II controller, which consists of a proportional-integral (PI) controller and a low pass filter (LPF), is as follows:

(25) $$G_{i}(s)=\frac{k(s+z)}{s(s+p)}.$$

The loop gain can be expressed as follows:

(26) $$L_{i}(s)=G_{i}(s)H_{i}(s)=\frac{k(s+z)}{s(s+p)}\frac{k_{s}K_{pwm}}{sL_{sh}}.$$

In this application case, the crossover frequency of a Type II controller is designed within the range of 1/4 to 1/10 of the switching frequency. This paper chooses the controller crossover frequency to be 1/10 of the switching frequency, the zero is designed at 1/4 of the crossover frequency, and the cut-off frequency of the LPF is designed to be 15 kHz:

(27) $$\omega_{i}=0.1\times 50k\times 2\pi =31416rad/s.$$

(28) $$z=\omega_{i}/4=7854rad/s.$$

(29) p=2π×15k=94248rad/s.

It follows that the gain of the plant at crossover frequency (Gain_Hi_) is as follows:

(30) $$\begin{array}{c} {\mathrm{Gain}}_{\mathrm{Hi}}=\frac{k_{s}K_{pwm}}{sL_{sh}}=\frac{2000}{j\omega }=0-j0.0637\\ \Rightarrow \left|{\mathrm{Gain}}_{\mathrm{Hi}}\right|=0.0637 \end{array}$$

The gain of the controller at crossover frequency (Gain_Gi1_) is as follows:

(31) $$\begin{array}{c} {\mathrm{Gain}}_{\mathrm{Gi}1}=\frac{\left(s+z\right)}{s(s+p)}=\frac{j\omega_{i}+7854}{j\omega_{i}(j\omega_{i}+94248)}=8.7535\times 10^{-6}-j5.5704\times 10^{-6}\\ \Rightarrow \left|{\mathrm{Gain}}_{\mathrm{Gi}1}\right|=1.0376\times 10^{-5} \end{array}$$

Then, the required gain for compensation at crossover frequency can be calculated:

(32) $$k=\frac{1}{\left|{\mathrm{Gain}}_{\mathrm{Hi}}\right|\times \left|{\mathrm{Gain}}_{\mathrm{Gi}1}\right|}=1.5139\times 10^{6}.$$

Finally, the transfer function is obtained as follows:

(33) $$G_{i}(s)=\frac{1.5139\times 10^{6}(s+7854)}{s(s+94248)}.$$

**Figure 4:**
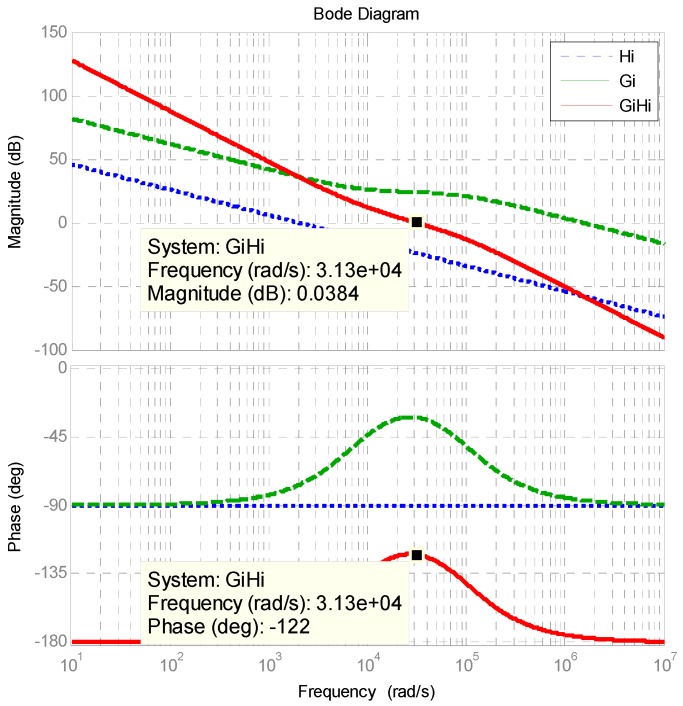
Bode plot of shunt active power filter (APF) inductor current control loop.

The designed *k_P_* and *k_I_* are 16.0633 and 2.5232956, respectively. Figure 4 shows the Bode plot of the controller and plant, where the designed phase margin is 58 degrees.

#### 3.3.3. Design of DC Link Voltage Controller

**Figure 5:**
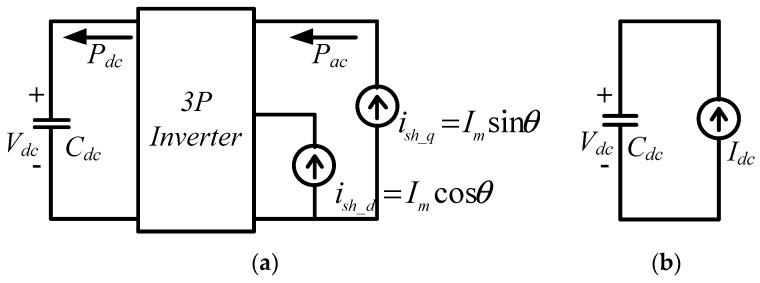
Equivalent circuits of the voltage control loop: (**a**) equivalent circuit under synchronous reference frame; (**b**) equivalent circuit on direct current (DC) side.

The DC link voltage control loop regulates the real power balancing between the alternating current (AC) and DC terminals of the three-phase inverter. By ignoring steady-state operating point, we can obtain equivalent small signal model of the voltage loop as shown in Figure 5.

The instantaneous AC power at the AC side can be defined as follows:

(34) $$P_{ac}=V_{m}\sin\theta *I_{m}\sin\theta +V_{m}\cos\theta *I_{m}\cos\theta ,$$

where *V_m_* and *I_m_* represent the maximum voltage and current under dq axes, respectively. According to trigonometric functions, Equation (34) can be simplified as follows:

(35) $$P_{ac}=V_{m}I_{m}.$$

Mapping the AC side signals onto the DC side and assuming that the inverter is lossless, we obtain the following:

(36) $$P_{ac}=P_{dc};$$

(37) $$V_{m}I_{m}=V_{dc}I_{dc}.$$

Then, we can obtain the relationship between the DC side current and the AC side current:

(38) $$I_{dc}=\frac{V_{m}}{V_{dc}}I_{m}=k_{dc}I_{m};$$

(39) $$C_{dc}\frac{dV_{dc}}{dt}=I_{dc}\Rightarrow V_{dc}=I_{dc}\frac{1}{sC_{dc}},$$

where *k_dc_* represents the conversion factor from AC side to DC side. According to Equations (38) and (39), we can obtain the transfer function of DC side voltage:

(40) $$\frac{V_{dc}}{I_{m}}=\frac{k_{dc}}{sC_{dc}},k_{dc}=\frac{V_{m}}{V_{dc}}.$$

**Figure 6:**
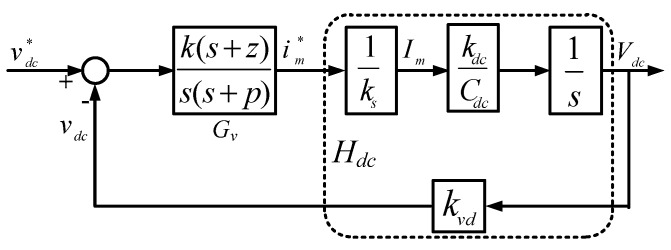
Block diagram of DC voltage loop type II controller.

According to the above derivations, we can obtain the block diagram of a DC link voltage control loop with a type-II controller as shown in Figure 6, where *k_vd_* and *k_s_* represent the sensing factors of DC voltage and AC current, respectively. Therefore, the transfer function of the DC voltage loop is as follows:

(41) $$H_{dc}(s)=\frac{k_{vd}k_{dc}}{k_{s}C_{dc}s}.$$

The transfer function of the Type II controller is defined as follows:

(42) $$G_{v}(s)=\frac{k(s+z)}{s(s+p)}.$$

It follows that the loop gain can be expressed as follows:

(43) $$L_{v}(s)=G_{v}(s)H_{dc}(s)=\frac{k(s+z)}{s(s+p)}\frac{k_{vd}k_{dc}}{k_{s}C_{dc}s}.$$

The main purpose of the Type II controller is to use an LPF to reduce possible interference affecting the DC link when the APF system compensates for PQ problems such as imbalance and harmonics in three-phase load currents. The crossover frequency is set at 1/500 of that of the current loop, the cut-off frequency of the LPF is set at 49 Hz, and the zero is designed at 1/5 of the crossover frequency of the DC loop:

(44) $$\omega_{v}=\omega_{i}\times 0.002=62.832rad/s;$$

(45) p=2π×49=307.8768rad/s;

(46) $$z=\omega_{v}/5=12.5664rad/s.$$

The gain of the plant at crossover frequency (Gain_Hdc_) can be calculated as follows:

(47) $$\begin{array}{l} {\mathrm{Gain}}_{\mathrm{Hdc}}=\frac{k_{vd}k_{dc}}{k_{s}C_{dc}s}=\frac{118.9}{j\omega_{v}}\\ =0-j1.8917\Rightarrow \left|{\mathrm{Gain}}_{\mathrm{Hdc}}\right|=1.8917 \end{array}$$

The gain of the controller at the crossover frequency (Gain_Gv1_) is as follows:

(48) $$\begin{array}{l} {\mathrm{Gain}}_{\mathrm{Gv}1}=\frac{\left(s+z\right)}{s(s+p)}=\frac{j\omega_{v}+12.57}{j\omega_{v}(j\omega_{v}+307.88)}\\ =0.003-j0.0013\\ \Rightarrow \left|{\mathrm{Gain}}_{\mathrm{Gv}1}\right|=0.0032 \end{array}$$

Then, the required gain compensation at the designed crossover frequency can be calculated by:

(49) $$k=\frac{1}{\left|{\mathrm{Gain}}_{\mathrm{Hdc}}\right|\times \left|{\mathrm{Gain}}_{\mathrm{Gv}1}\right|}=165.1953.$$

Finally, the transfer function of the voltage controller is obtained:

(50) $$G_{v}(s)=\frac{165.1953(s+12.5664)}{s(s+307.8768)}.$$

**Figure 7:**
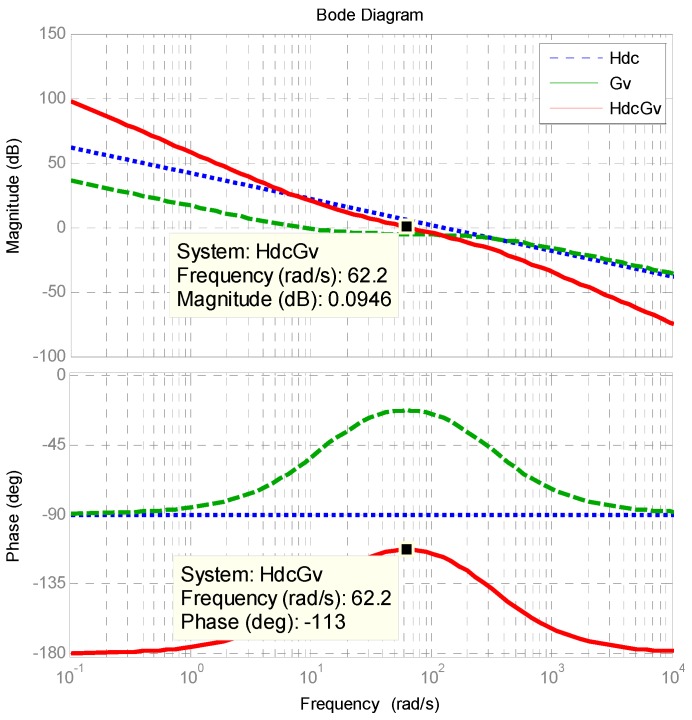
Bode plot of shunt APF inductor DC link voltage control loop.

The designed *k_P_* and *k_I_* are 0.5286 and 0.0001329397, respectively. Figure 7 shows the Bode plot of the controller and plant, where phase margin is 67 degrees.

#### 3.3.4. Load Current Compensation Signals of APF

**Figure 8:**
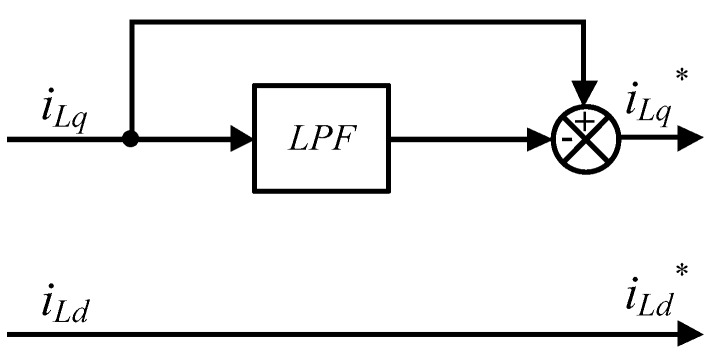
The direct-quadrature axis currents compensation signals of the APF.

Using the SRF conversion technique, distorted and unbalanced three-phase load currents can be expressed as follows:

(51) $$\left[\begin{array}{c} i_{Ld}\\ i_{Lq} \end{array}\right]=\left[\begin{array}{c} {\bar{i}}_{Ld}\\ {\bar{i}}_{Lq} \end{array}\right]+\left[\begin{array}{c} {\tilde{i}}_{Ld}\\ {\tilde{i}}_{Lq} \end{array}\right],$$

where *i_Ld_* and *i_Lq_* represent dq-axis load currents, ${\bar{i}}_{Ld}$ and ${\bar{i}}_{Lq}$ represent dq-axis load currents with the fundamental frequency, and ${\tilde{i}}_{Ld}$ and ${\tilde{i}}_{Lq}$ represent the dq-axis components that require compensation. In order to obtain the compensation signals of the active current (q axis) i_Lq_^*^, *i_Lq_* is firstly filtered with an LPF and then subtracted from q-axis current feedback signal (*i_Lq_*), while the compensation signals of the reactive current (d axis) i_L__d_^*^ equals the whole d-axis current feedback signal (*i_Ld_*), as shown in Figure 8.

### 3.4. Complete System of GaN-Based Shunt APF

**Figure 9:**
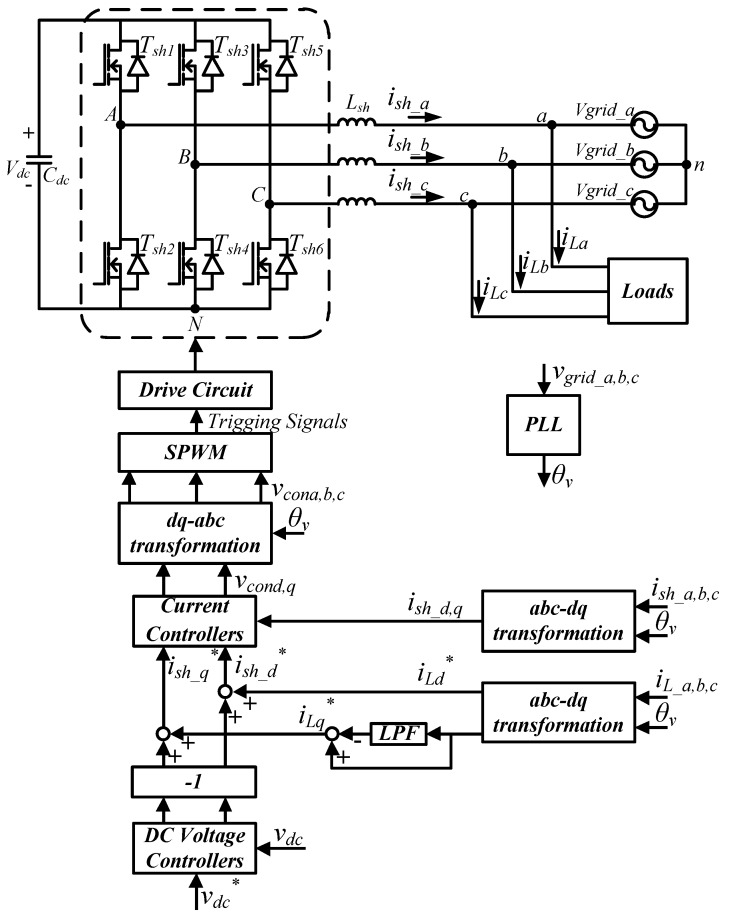
The circuit configuration of gallium nitride (GaN)-based shunt APF system and the block diagram of the control scheme.

According to Figure 8, DC link voltage controller, and inductor current controllers, we can obtain the circuit configuration of the proposed GaN based three-phase APF system with the block diagram of complete control architecture, as shown in Figure 9. 

## 4. Simulation Study and Results

**Figure 10:**
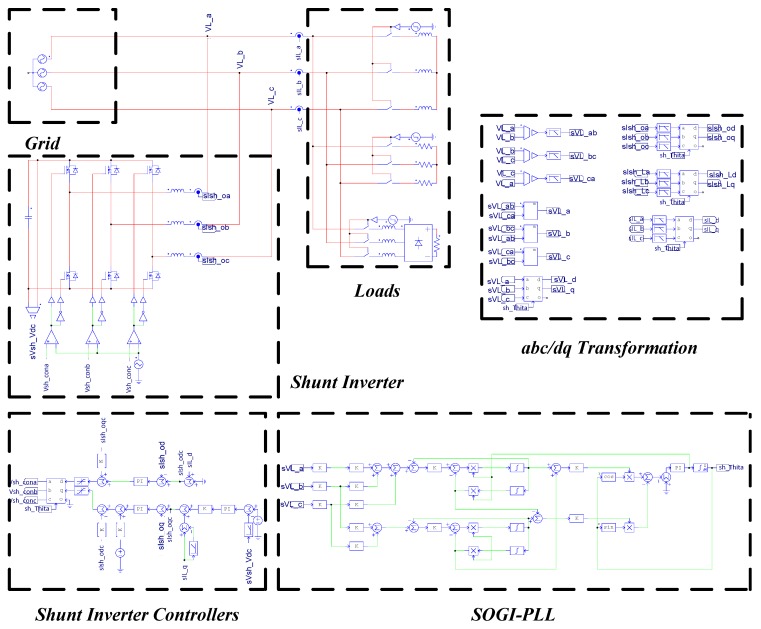
Powersim (PSIM) simulation model of the proposed APF system.

With the design presented in the previous section, the proposed Gan-based shunt-type APF is tested for an integrated compensation of multiple power quality problems, including current harmonics, load current imbalance, and reactive currents. Powersim (PSIM) software is used to perform the simulation case of the abovementioned comparison tasks. The PSIM simulation model is shown in Figure 10.

### 4.1. Simulation Scenario

**Figure 11:**
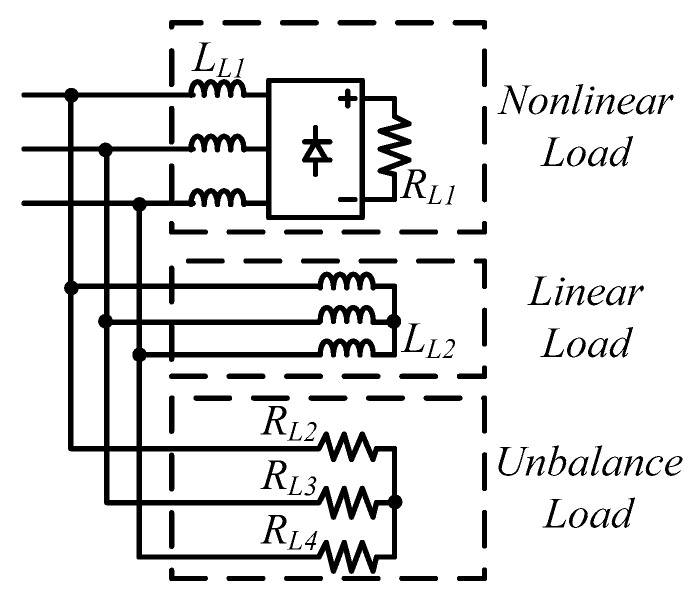
Load condition in simulated scenario.

**Figure 12:**
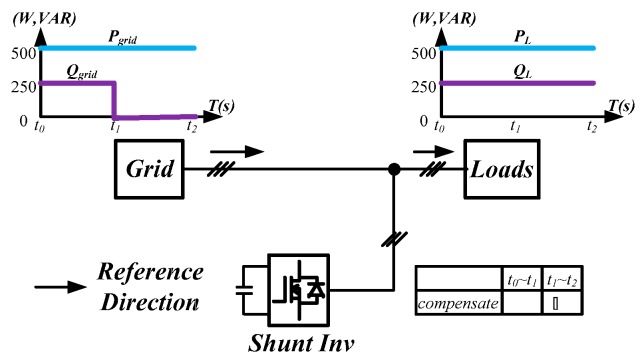
Schematic diagram of simulated scenario.

**Figure 13:**
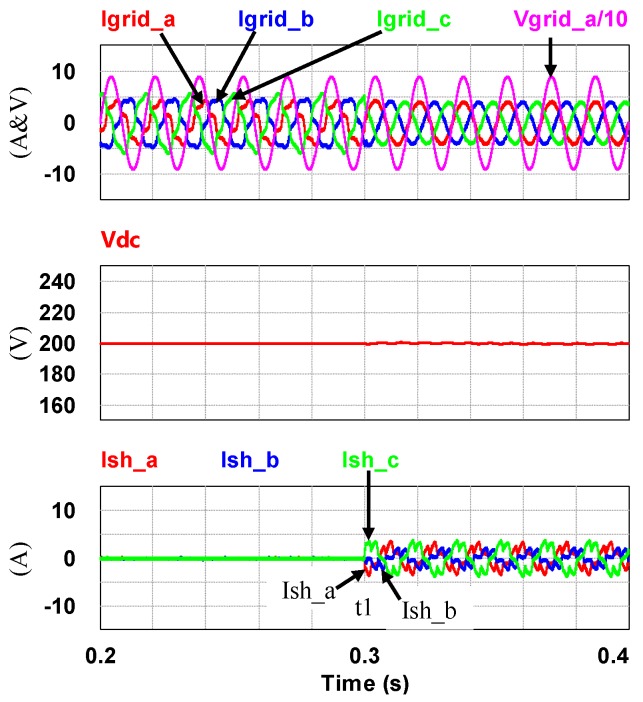
The grid-side phase-a voltage and three-phase currents/DC link voltage/shunt APF three-phase currents (t_0_–t_2_).

**Figure 14:**
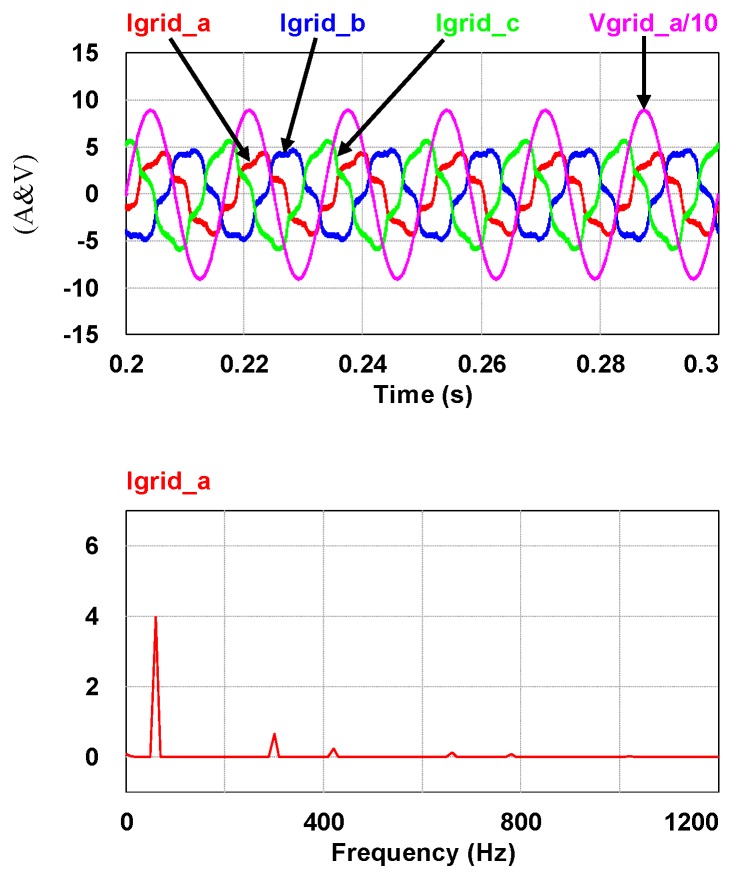
Before t_1_: the grid phase-a voltage and three-phase currents/the fast Fourier transform (FFT) waveform of the grid phase-a current.

**Figure 15:**
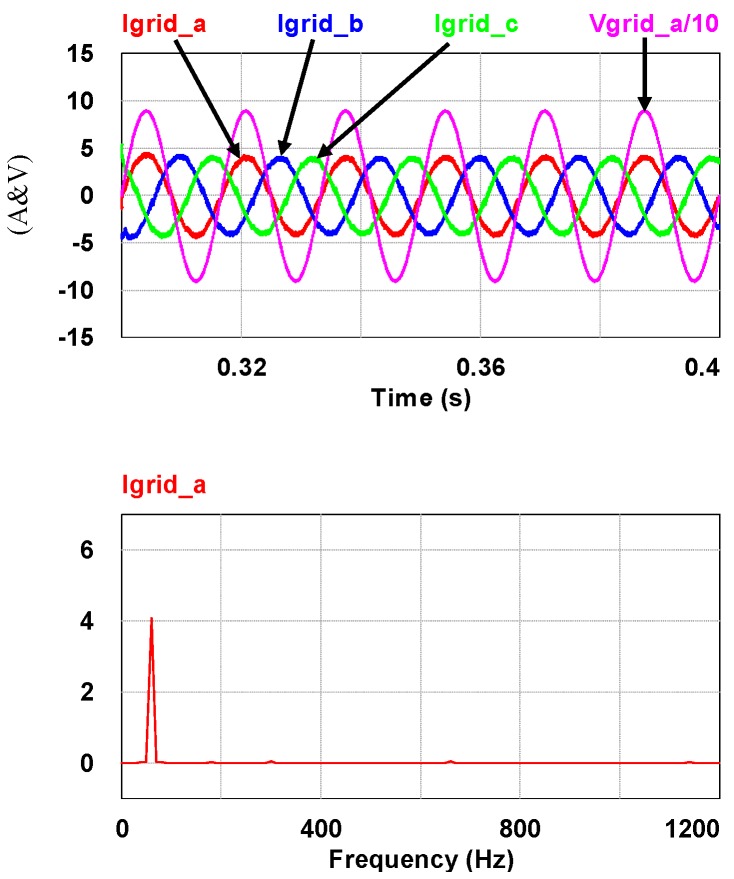
After t_1_: the grid phase-a voltage and three-phase currents/FFT waveform of the grid phase-a current.

**Figure 16:**
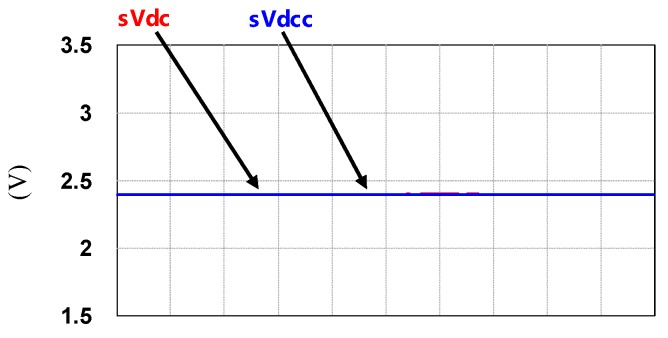
Shunt APF DC link voltage command and feedback signals (t_0_–t_2_).

**Figure 17:**
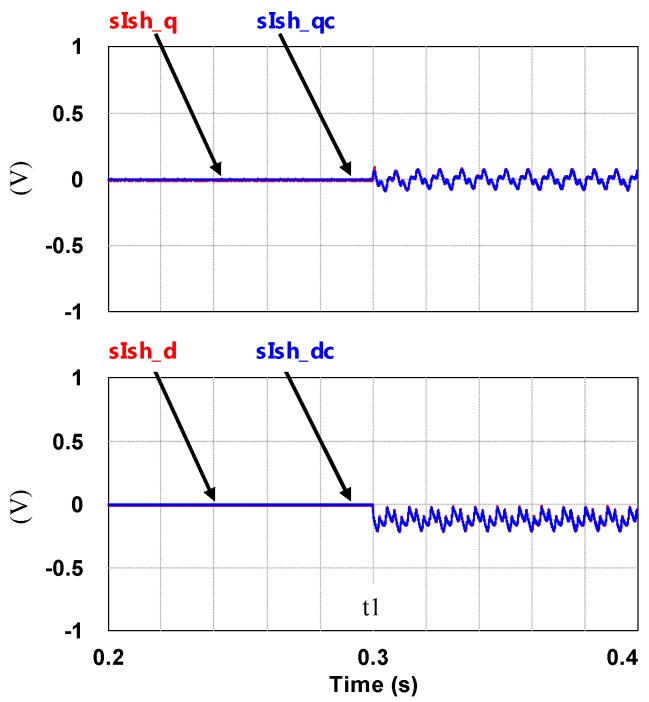
Shunt APF dq-axis current commands and feedbacks (t_0_–t_2_).

To demonstrate the performance of the proposed controllers, the integrated compensation for multiple load current quality problems with APF is simulated. In this case, the three-phase load bank consists of a balanced reactive load, an unbalance resistive load, and a non-linear load, as shown in Figure 11. Table 1 shows the detailed values of the loads used. At first (t_0_–t_1_), the shunt APF, connected to a three-phase power grid with the line to line voltage of 110 V, 60 Hz, adjusts the DC link voltage to 200 V and the compensation function is not activated; at t_1_, compensation is activated to achieve a set of balanced grid currents, zero distortion, and unit power factor (PF), as shown in Figure 12. Figure 13, Figure 14, Figure 15, Figure 16 and Figure 17 show the corresponding simulation results, and Table 2 shows root-mean-square (RMS) currents and total harmonic distortion (THD) data before and after compensation.

## 5. Hardware Implementation and Test Results

**Figure 18:**
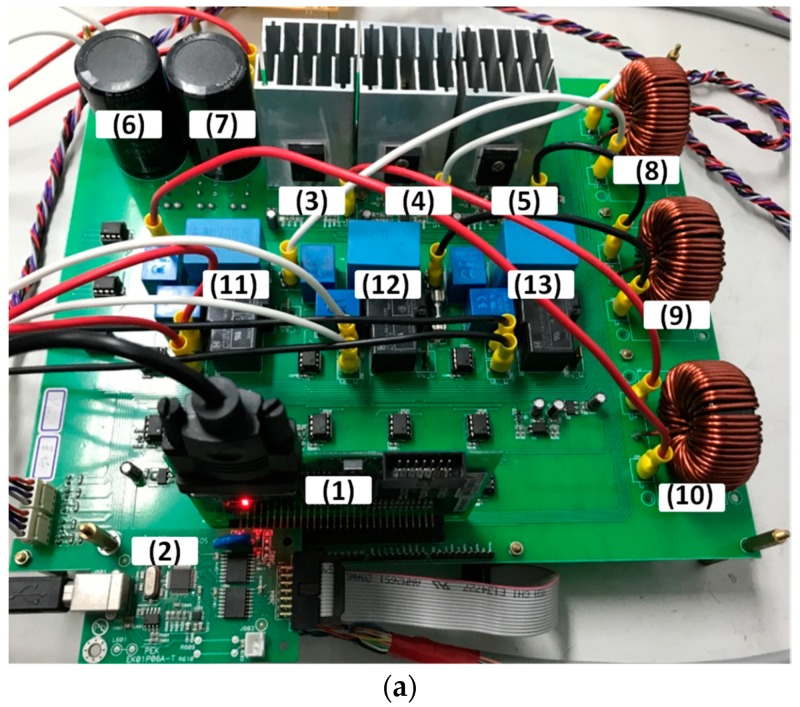

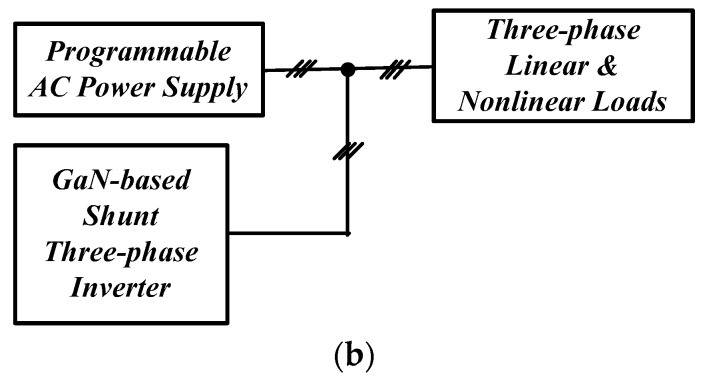
(**a**) Photo of GaN-based three-phase APF hardware; (**b**) schematic of the hardware test and system.

**Figure 19:**
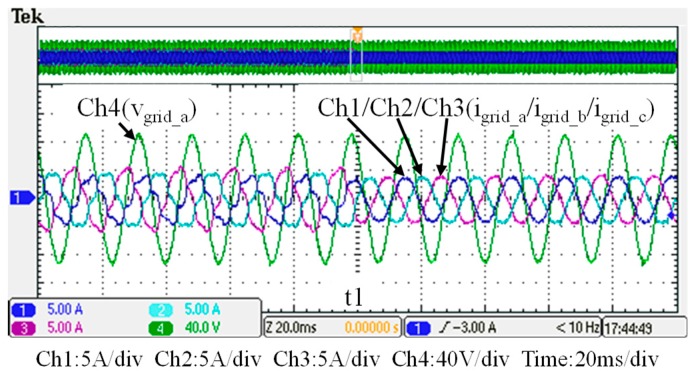
Grid phase-a voltage and three-phase currents (t_0_–t_2_).

**Figure 20:**
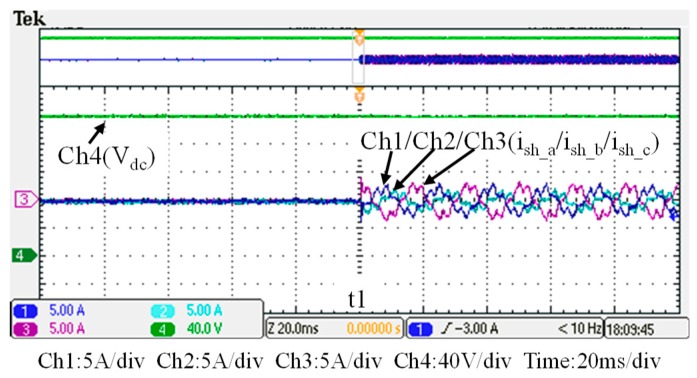
DC link voltage and the shunt APF three-phase currents (t_0_–t_2_).

**Figure 21:**
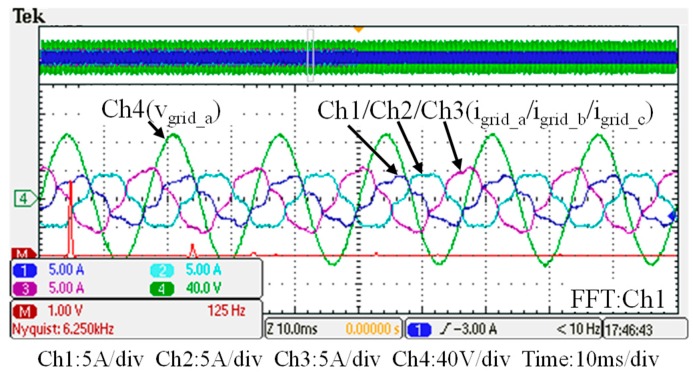
Before t_1_: the grid phase-a voltage and three-phase currents and the fast Fourier transform (FFT) waveform of the grid phase-a current.

**Figure 22:**
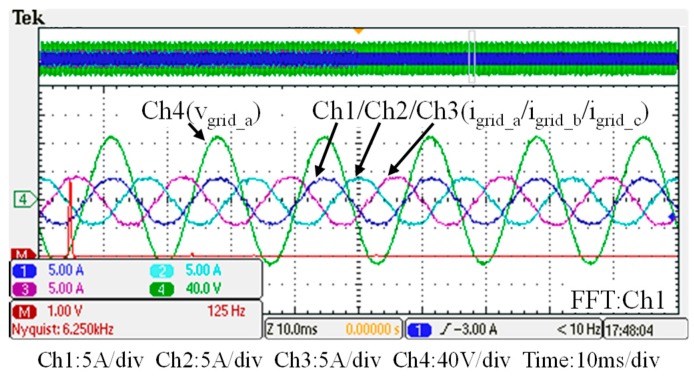
After t_1_: the grid phase-a voltage and three-phase currents and FFT waveform of the grid phase-a current.

**Figure 23:**
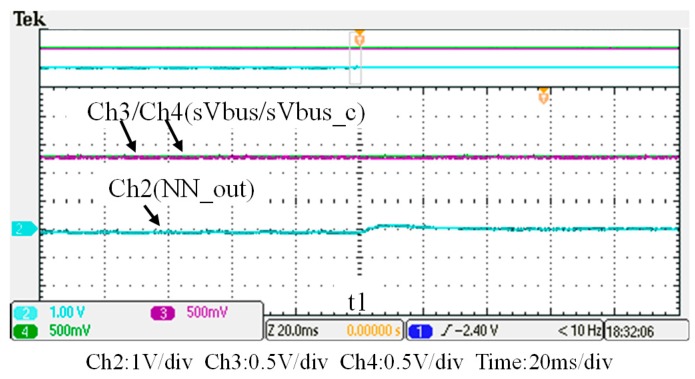
The command and feedback signals of DC link voltage and the proportional-integral (PI) controller output signal (t_0_–t_2_).

**Figure 24:**
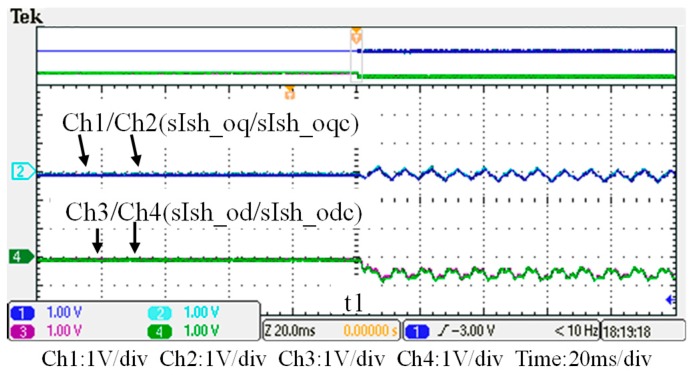
The shunt APF dq-axis current commands and feedbacks (t_0_–t_2_).

To verify the performance of the proposed GaN-based APF, this section presents the implementation of APF hardware prototype for verification and analysis based on the scenario arranged in the simulation case stated in the previous section. The photograph of the constructed GaN-based APF prototype is shown in Figure 18, where the numbered devices are listed in Table 3. A programmable three-phase AC power supply is adopted to emulate the grid voltage. The Texas Instruments (TI) microcontroller, TMS320F28335 (Texas Instruments, Dallas, TX, USA), is used to provide efficiency and flexibility in controller design. The system parameters and conditions of the experimental tests and measurement scenarios are the same as that used in the previous simulation case presented in Section 4.1. Figure 19, Figure 20, Figure 21, Figure 22, Figure 23 and Figure 24 show a set of test results; Figure 19 shows the waveforms of measured phase-a voltage and three-phase currents of the grid from t_0_ to t_2_. Figure 20 shows the DC link voltage and the output three-phase currents of the shunt APF from t_0_ to t_2_. The related waveforms of grid phase-a voltage and three-phase currents and the fast Fourier transform (FFT) of the grid phase-a current before the before and after the APF is activated are shown in Figure 21 and Figure 22, respectively. As can be seen in Figure 22, after the APF is activated the unbalanced and distorted currents have been well compensated and the current is in phase with the grid voltage achieving the control objective of unity power factor. To demonstrate the performance of the designed controllers, Figure 23 shows the command and feedback signals of DC link voltage and the PI controller output signals. The dq-axis current commands and feedback signals are shown in Figure 24. To provide a set of quantitative results, Table 4 shows the measured RMS currents and calculated THD data before and after compensation. In the stage of hardware construction and tests, the system efficiencies at different switching frequencies are also explored. The arrangement of the test scenario and the detailed results are presented in the next section. 

## 6. Discussion

### 6.1. The Analysis of System Efficiency

**Figure 25:**
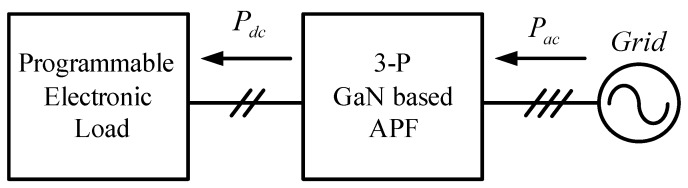
The system block diagram of the efficiency tests.

**Figure 26:**
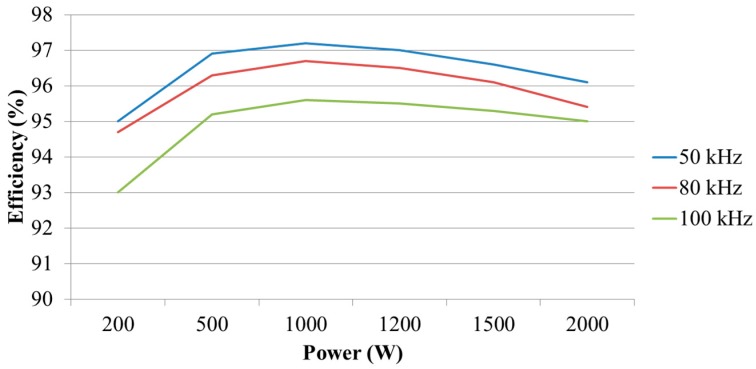
Efficiencies of GaN-based shunt APF system at different switching frequencies.

In this paper, the system efficiencies at different switching frequencies (50 kHz, 80 kHz, and 100 kHz) and under different load levels are explored. Figure 25 shows the system block diagram of the efficiency tests. In this test, the AC terminals of the proposed three-phase GaN-based APF are connected to the three-phase power grid having the line to line voltage of 110 V and its DC terminal voltage is regulated at the designed 200 V by the proposed voltage controller of the APF. For testing the APF efficiencies under different load levels, a programmable electronic load is connected to the DC terminal of the APF. By setting different *P_dc_* and measuring the corresponding *P_ac_,* the efficiency at a specific power level and switching frequency can be readily calculated. In this paper, three switching frequencies, i.e., 50, 80, and 100 kHz were tested. The calculated results are graphically shown in Figure 26. As can be seen in Figure 26, the maximum efficiency appears at about 50% of the rated load and it is found that when the switching frequency increases the efficiency decreases. This is mainly due to the increase in switching losses. 

In the aspect of the efficiency comparison with different technologies, it is indeed difficult to establish a fair comparative basis due to some system constrains, e.g., the switching technique used, system capacities, quality of components used, and the control functions designed for the circuits. To provide a set comparative result, three recently published technical papers [16,17,18] using different technologies are summarized in Table 5. 

### 6.2. The Thermographic Analysis of the System

**Figure 27:**
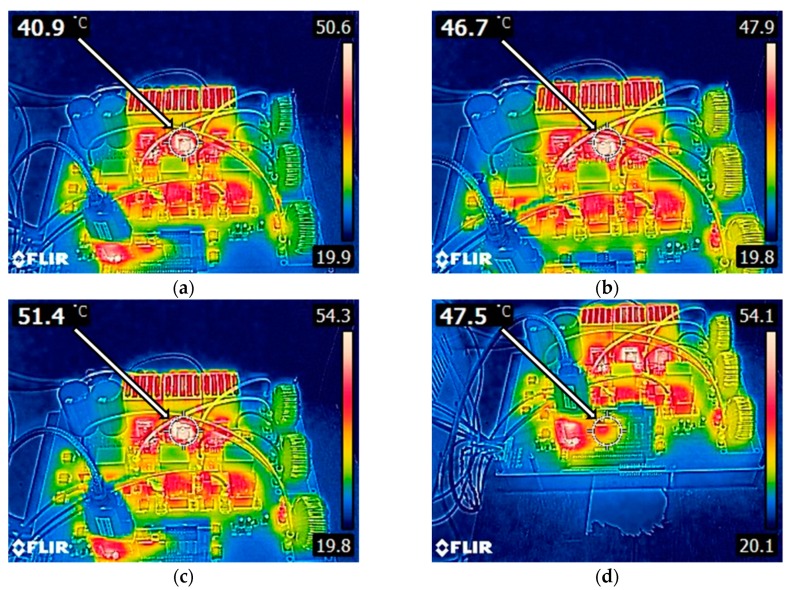

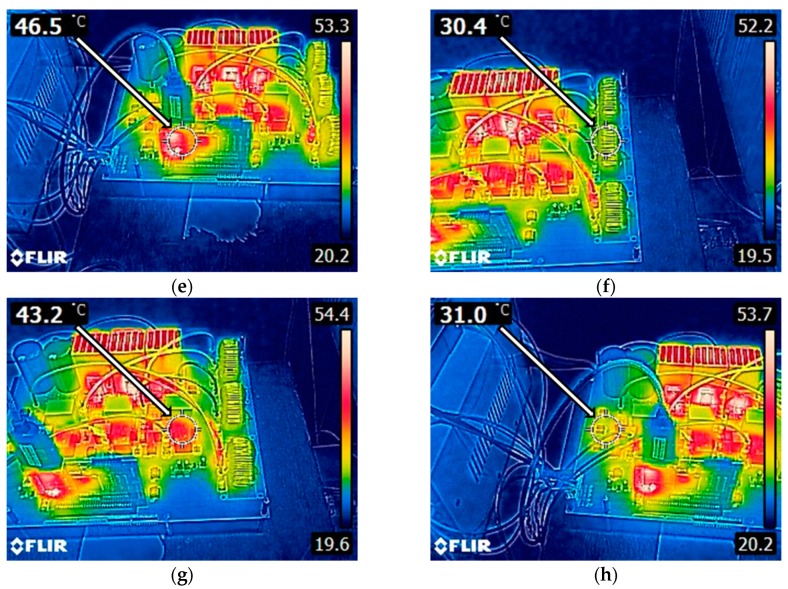
Thermographic photos of the proposed GaN-based three-phase APF prototype: (**a**) TPH3207 device switching at 50 kHz; (**b**) TPH3207 device switching at 80 kHz; (**c**) TPH3207 device switching at 100 kHz; (**d**) DSP; (**e**) communication interface; (**f**) inductors; (**g**) relay; (**h**) signal processing integrated circuits (ICs).

Figure 27 shows a set of thermographic photos (using FLIER-E63900, T198547, E4) of the proposed GaN-based three-phase APF prototype operating under the rated capacity of 2 kW with different switching frequencies. As can be seen in the photos, the temperature of the switching devices increases as the switching frequency increases and the temperature of capacitors and the circuit board remain under 25 °C. Table 6 shows the summary of the recorded temperature data gathered from the presented thermographic photos. Based on the results of thermographic analysis, it has been found that the greatest losses are located at the six power-switching devices and the three relays which are designed for ensuring a successful synchronizing control with the power grid to which the proposed APF is connected. It should be noted that in practice, these relays can be removed after the overall control system of the APF has been properly tuned. This means that the maximum efficiency of the proposed GaN-based APF can be further improved.

### 6.3. Related Technical Issues

As mentioned previously, the proposed GaN-based three-phase APF circuit prototype is demonstrated for the first time. There are some technical issues to be further improved. These include: (1) the driving circuits can be improved to achieve higher switching frequency and to reduce the size of inductors; (2) the three relays can be removed or replaced with new devices having better quality in conduction losses to increase the overall system efficiency; (3) the layout of the circuit can be improved to reduce the noise level in current- and voltage-sensing mechanisms. It is important to note that the noise level in sensing signals constitutes the operating limits of the switching frequency of the proposed GaN-based APF circuit.

## 7. Conclusions

It has been expected that the mass application of RE-based distributed generation (DG) microgrids or smart grids, and various static and dynamic nonlinear loads is the future trend of development in power systems. To ensure an acceptable PQ level, the development of advanced PQ compensation schemes using new technologies in terms of power-switching devices and state-of-the-art control algorithms is a significant task. In this aspect, this paper has demonstrated, for the first time, a shunt-type GaN HEMTs-based three-phase APF controlled by DSP systems and type II controllers to achieve simultaneous compensation for current harmonics, load imbalance, and reactive currents. Based on the results obtained from simulation and the hardware tests, the proposed 2-kVA GaN-based three-phase shunt APF prototype with digitally integrated control scheme is able to achieve satisfactory compensation results in improving system-wide load current quality of a complex load network consisting of distorted, non-linear and unbalanced loads. In this study, the TPH3207 power switching devices and Si8271 driving integrated circuits (ICs) are successfully adopted. It has been found that GaN HEMTs provide superior performance to conventional Si-based power switches in terms of switching frequency, temperature feature and system efficiency. To further evaluate the system performance of the constructed GaN-based circuit prototype, in terms of efficiency, hotspot distribution and power losses in components, a thermographic analysis has been carried out. Results and discussions for improving the proposed implementation scheme have been presented. With the proposed APF operating at 50 kHz switching frequency, the THD and UR of the three-phase grid currents can be greatly reduced. The best THD improvement recorded is from 20.23% to 4.15% and UR is from 14.83% to 2.13% and the highest system efficiency of 97.2% has been achieved. For future research works, better circuit components and GaN HEMT driving methods based on a bootstrap design can be used for achieving higher switching frequency and better system performance.

## Figures and Tables

**Table 1:** Load parameters for simulation scenario.

Nonlinear Load			Linear Load	Unbalanced Load		
R_L1_	L_L1_		L_L2_	R_L2_	R_L3_, R_L4_	
50 Ω		6 mH × 3	152 mH × 3	∞ Ω		50 Ω

**Table 2:** Root-mean-square (RMS) currents and total harmonic distortion (THD).

Power Grid Currents and Unbalance Ratio (UR)			
**Item**	**Without APF** **(t_0_–t_1_)**		**With APF** **(t_1_–t_2_)**
**i_grid_a_ (A)**	2.9	2.84	
**i_grid_b_ (A)**	3.42	2.87	
**i_grid_c_ (A)**	3.87	2.89	
**UR (%)**	14.91	0.94	
**THD**			
**Item**	**Without APF** **(t_0_–t_1_)**	**With APF** **(t_1_–t_2_)**	
**THD (i_grid_a_) (%)**	19.18	3.91	
**THD (i_grid_b_) (%)**	15.56	3.94	
**THD (i_grid_c_) (%)**	13.89	3.94	

**Table 3:** Devices in Figure 18a.

Number	Device	Value/Part Number
(1)	Microcontroller	TMS320F28335
(2)	Interface circuit of the microcontroller	N/A
(3)–(5)	GaN HEMT pairs	TPH3207
(6) and (7)	DC link capacitors	680 mF/450 V
(8)–(10)	Filter inductors	0.5 mH
(11)–(13)	Filter capacitors	10 μF/300 V

**Table 4:** RMS currents and THD.

Power Grid Currents and Unbalance Ratio (UR)		
**Item**	**Without APF (t_0_–t_1_)**	**With APF (t_1_–t_2_)**
**i_grid_a_ (A)**	2.93	2.91
**i_grid_b_ (A)**	3.43	2.94
**i_grid_c_ (A)**	3.95	3.02
**UR (%)**	14.83	2.13
**THD**		
**Item**	**Without APF (t_0_–t_1_)**	**With APF (t_1_–t_2_)**
**THD (i_grid_a_) (%)**	20.23	4.15
**THD (i_grid_b_) (%)**	16.57	4.08
**THD (i_grid_c_) (%)**	15.48	4.05

**Table 5:** Performance comparison of different technologies.

Paper	Switching Device	Function	Power	Switching Frequency	Efficiency
[16]	IGBT	Motor drive	8 kW	20 kHz	95.5%
[17]	MOSFET	Motor drive	1.5 kW	15 kHz	92%
[18]	SiC	Electric vehicle	8.8 kW	50 kHz	97%
proposed	GaN	Active power filter	2 kVA	50 kHz	97.2%
				80 kHz	96.7%
				100 kHz	95.6%

**Table 6:** Summary of operating temperatures of individual devices.

Sensed Object	Operating Temperature (°C)
TPH3207 @ 50kHz switching frequency	40.9
TPH3207 @ 80kHz switching frequency	46.7
TPH3207 @ 100kHz switching frequency	51.4
DSP	47.5
Communication interface	46.5
Inductors	30.4
Relays	43.2
Signal processing integrated circuits (ICs)	31.0

## References

[1] Gayatri M.T.L., Parimi A.M., Pavan Kumar A.V. (2018). A review of reactive power compensation techniques in microgrids. Renew. Sustain. Energy Rev..

[2] Naderi Y., Hosseini S.H., Zadeh S.G., Mohammadi-Ivatloo B., Vasquez J.C., Guerrero J.M. (2018). An overview of power quality enhancement techniques applied to distributed generation in electrical distribution networks. Renew Sustain. Energy Rev..

[3] Kumar R., Bansal H.O. (2018). Shunt active power filter: Current status of control techniques and its integration to renewable energy sources. Sustain. Cities Soc..

[4] Baimel D. (2019). Implementation of DQ0 control methods in high power electronics devices for renewable energy sources, energy storage and FACTS. Sustain. Energy Grids.

[5] Swain S., Subudhi B. (2018). Grid integration scheme for a three phase single stage photovoltaic system employing EKF. IFAC-PapersOnLine.

[6] Chauhan S., Singh B. (2019). Grid-interfaced solar PV powered electric vehicle battery system with novel adaptive digital control algorithm. IET Power Electron..

[7] Zhai H., Zhuo F., Zhu C., Yi H., Wang Z., Tao R., Wei T. (2020). An optimal compensation method of shunt active power filters for system-wide voltage quality improvement. IEEE Trans. Ind. Electron..

[8] Badoni M., Singh A., Singh V.P., Tripathi R.N. (2018). Grid interfaced solar photovoltaic system using ZA-LMS based control algorithm. Electr Power Syst. Res..

[9] Prasad V., Jayasree P.R., Sruthy V. (2018). Active power sharing and reactive power compensation in a grid-tied photovoltaic system. Mater. Today: Proc..

[10] Viswan V. (2018). A review of silicon carbide and gallium nitride power semiconductor devices. IJRESM.

[11] Jones E.A., Wang F., Costinett D. (2016). Review of commercial GaN power devices and GaN-based converter design challenges. IEEE J. Emerg. Sel. Topics Power Electron..

[12] Spaziani L., Lu L. Silicon, GaN and SiC: There’s room for all: An application space overview of device considerations. Proceedings of the 2018 IEEE 30th International Symposium on Power Semiconductor Devices and ICs (ISPSD).

[13] Ma C.T., Gu Z.H. (2019). Review of GaN HEMT Applications in Power Converters over 500 W. Electronics.

[14] Gwóždž M. Active power filter with sigma-delta modulator in control section. Proceedings of the 2017 19th International Conference on Electrical Drives and Power Electronics (EDPE).

[15] Otero-De-Leon R., Liu L., Bala S., Manchia G. Hybrid active power filter with GaN power stage for 5kW single phase inverter. Proceedings of the 2018 IEEE Applied Power Electronics Conference and Exposition (APEC).

[16] Uğur M., Saraç H., Keysan O. Comparison of Inverter Topologies Suited for Integrated Modular Motor Drive Applications. Proceedings of the 2018 IEEE 18th International Power Electronics and Motion Control Conference (PEMC).

[17] Mahmoudian M., Gitizadeh M., Rajaei A.H., Tehrani V.M. (2019). Common mode voltage suppression in three-phase voltage source inverters with dynamic load. IET Power Electron..

[18] Fu Y., Li Y., Huang Y., Bai H., Zou K., Lu X., Chen C. (2019). Design methodology of a three-phase four-wire EV charger operated at the autonomous mode. IEEE Trans. Transp. Electr..

